# Peripheral ameloblastoma: A case report

**DOI:** 10.4317/jced.56757

**Published:** 2020-06-01

**Authors:** Ersin Ülker, Tuğrul Kirtiloğlu, Burcu Taban

**Affiliations:** 1 Research Assistant, Department of Periodontology, Faculty of Dentistry, Ondokuz Mayis University, Samsun, Turkey; 2 Associated Proffessor, Department of Periodontology, Faculty of Dentistry, Ondokuz Mayis University, Samsun, Turkey

## Abstract

**Background:**

Ameloblastoma is a rare tumor which develops from odontogenic epithelium and its remnants and it occurs in the jaws. Peripheral ameloblastomas are rare and benign extraooseous ameloblastomas which effects soft tissues. This case report declares a peripheral ameloblastoma which is a rare type of ameloblastoma.

**Material and Methods:**

34 year old female patient referred with a complaint of a gingival growth at right lower premolar area. A firm and granular surfaced gingival growth with the color of pink and red and having 1.5x1 cm sizes was observed at the mentioned area. With an incision from lower right second incisor tooth to lower right second molar tooth a flap from bone was made and lesion was excised. After then specimen was submitted to histopathologic examination. After clinical, radiological and pathological examinations lesion was described as peripheral ameloblastoma.

**Results:**

At the control examination after three months of excision there was no recurrence and patieant has no complaint.

**Conclusions:**

Although reccurens rate of peripheral ameloblastomas are low, long-term follow-ups are suggested Patient was informed about the importance of regular controls for early diagnosis of possible reccurenses and regular controls were made during one year after excision.

** Key words:**Peripheral ameloblastoma, gingiva, gingival hyperplasia, gingival lesion, alveolar mucosa, extraosseous.

## Introduction

Ameloblastoma is a rare tumor which develops from odontogenic epithelium and its remnants and it occurs in the jaws (1,2). According to the classification of WHO there are 4 clinical types of ameloblastomas; solid, desmoplastic, unicystic and peripheral (3). But currently classification of ameloblastomas is simplified and ameloblastomas are seperated into two types as unicystic ameloblastomas and peripheral/extraosseous ameloblastomas (4). Peripheral ameloblastomas are rare and benign extraooseous ameloblastomas which effects soft tissues and they were introduced to literature first by Kuru but first true description of them was made by Stanley and Krogh (5,6).

Peripheral ameloblastomas are rare cases which have %1.3-10 ratio among all ameloblastomas (3). This case report declares a peripheral ameloblastoma which is a rare type of ameloblastoma.

## Case Report

**Figure 1:**
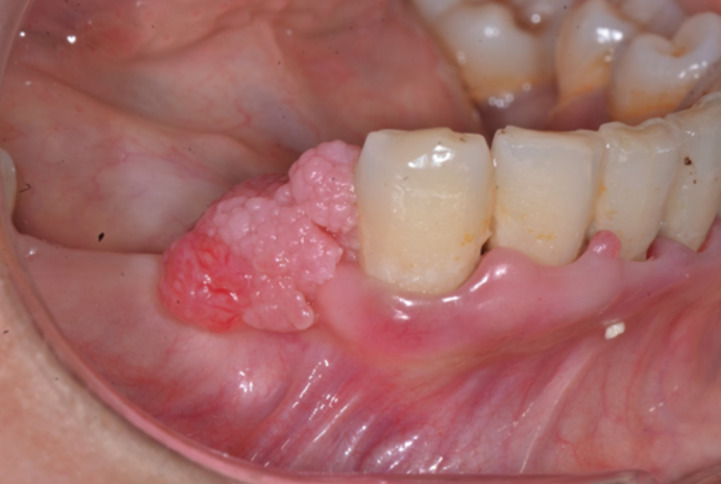
Intraoral view of the lesion.

**Figure 2:**
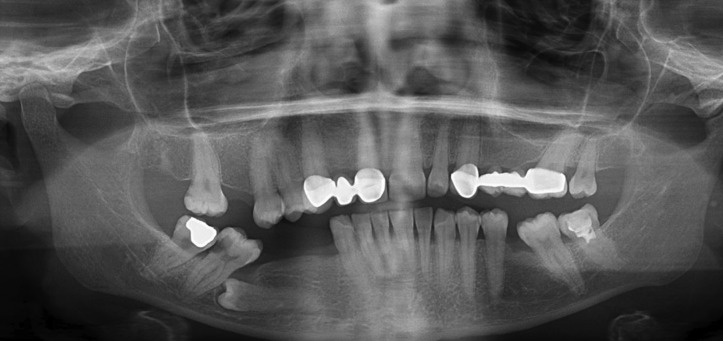
Extraoral panoramic radiography of the patient.

34 year old female patient referred to Ondokuz Mayıs University, Faculty of Dentistry, Department of Periodontology with a complaint of a gingival growth at right lower premolar area. Patient reported that she realized the mentioned growth first one month ago and she has no pain or bleeding complaint. With the anamnesis of the patient it has been learned that patient has no systemic disease and no drug use. Patient also reported no use of cigarette and alcohol. At the extraoral examination there was no extraoral finding like swelling or lymphadenopathy. At the intraoral examination it was seen that lower right first premolar, second premolar and first premolar teeth were lost at the mentioned area and a firm and granular surfaced gingival growth which seems like pyogenic granulom/ giant cell granulom with the color of pink and red and having 1.5x1 cm sizes was observed over the edentate alveolar cret where first and second premolar teeth should be (Fig. 1). There was no pain at the palpation of the lesion. With the extraoral panoramic radiography an impacted premolar teeth was seen at the mentioned area (Fig. 2). But there was no change at the bone structure at the exact location of the gingival growth.

**Figure 3:**
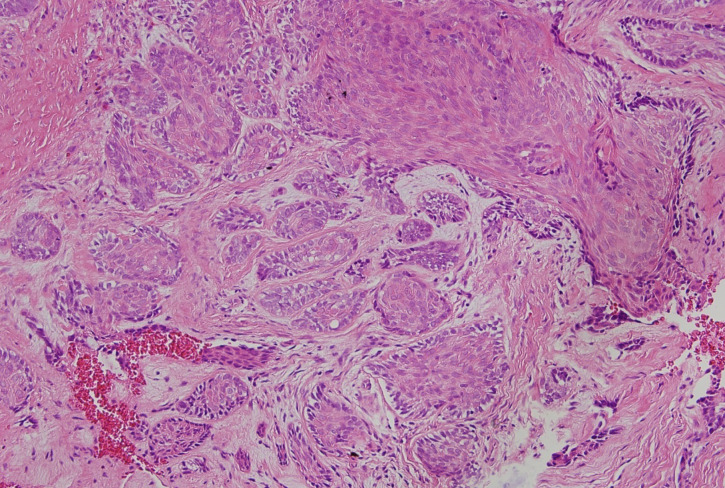
Micro maginification of the specimen (HEX200).

With an incision from lower right second incisor tooth to lower right second molar tooth a flap from bone was made and lesion was excised. After then specimen was submitted to histopathologic examination. At the macro magnification (HEx40) basoloid cell islands was observed at the loose connective tissue which shows proliferation to beneath of ceratinized stratified flat epithelium. And at the micro magnification (HEx200) basoloid cell islands which shows reverse palizades at their periphery were observed and pathologic diagnosiz was made as ameloblastoma (Fig. 3). After clinical,pathological and radiological examinations lesion was described as peripheral ameloblastoma. At the control examination after three months of excision there was no recurrence and patient has no complaint. Additionally patient was informed about the importance of regular controls for early diagnosis of possible reccurenses and regular controls were made during one year after excision.

## Discussion

Peripheral ameloblastomas are generally common at one location. Only Hernandez *et al.* reported only one case that located at two diffrent locations at the same time.7,8

 Peripheral ameloblastomas usually occur at lower jaw premolar area, followed by lower anterior and maxillar tuber areas. These cases can be seen at the ages raging from 9 to 92 and mean age rewiewed is 52.1. Peripheral ameloblastomas are more common for male patients then female patients (%65) (9). In thıs case compatible to literature lesion was at lower premolar area but the patient was younger and female.

 Peripheral ameloblastomas can be drawn with lots of clinical situations and they can be noticed incidentally at routine dental examination (10). In this case patient referrred to our department with an evident complaint because of noticing abnormal growth of her gingiva and feeling irritation during occlusion because the contact of lower gingiva to upper jaw .

 Periphreal ameloblastomas are painless, sessile, firm and granular or pebbly like surfaced exophytic growths and their clinical appearance can be interefered with many different clinical situations like pyogenic granuloma, giant cell granuloma, inflammatory fibrouse hyperplasia related to prosthesis and basal cell carsinoma (11,9,12). For this reason usually final diagnosis are made after histopathologic examinations like our case.

Generally Peripheral ameloblastomas do not penetrate to bone structure and lesions do not affect the cortical bone which they have been located on (9). In our case compatible with these informations it wasn’t seen a change or an invasion at cortical bone both during operation and radiologic examination. Only at the distal surface of the canine tooth there was a periodontal pocket formation and vertical bone defect related to plaque and subgingival calculus. After making debrisman process at mentioned area during operation both clinical and radiological recovery was observed at follow up examinations.

 Impacted tooth relation is a common situation for unicystic ameloblastomas according to literature but only one case that reporting peripheral ameloblastoma with an impacted tooth was found at the literature search (13,7). In this case an impacted tooth was seen placed at a close location to lesion at panoramic radiography but it was not thought to be in relation with the lesion. Fort the treatment of peripheral ameloblastomas a surgical excision reaching to sound tissues are advised. Radiotherapy or resection are not adviced because of being considered an overtreatment (9,14). Although reccurens rate of peripheral ameloblastomas are low, long-term follow-ups are suggested (15). It was reported that a benign looking peripheral ameloblastoma was reccurated as an ameloblastic carsinoma (16). Additionally a peripheral ameloblastoma that metastates and a recurrence of a peripheral ameloblastoma which shows displasia was reported too (17,18). Under the light of these informations the importance and necessity of long term regular controls is understood.
